# Role of Physical Exercise, Education and Work Related Measures with the Longevity of Work in Older Population in United States

**DOI:** 10.4236/aar.2017.61001

**Published:** 2016-12-13

**Authors:** Vatsalya Vatsalya, Antero Heloma, Gulshan L. Khanna, Kan V. Chandras, Robert C. Karch

**Affiliations:** 1 College of Arts and Sciences, American University, Washington DC, USA; 2 National institute of Health and Welfare, Helsinki, Finland; 3 Manav Rachna International University, Aravalli Campus, Faridabad, India; 4 Behavioral Sciences, Fort Valley State University, Fort Valley, GA, USA

**Keywords:** Aging, Health, Duration, Measures, Work-Life

## Abstract

Growth of older population in United States (US) raises concerns for evaluation of health indices that could sustain their workability. This study aimed to characterize the association of health practices used by older working population and measures of quality and duration of their work. Forty (40) non-treatment seeking healthy working individuals residing within United States within 22 - 75 years of age were included in this study. Data were collected from the Customized Employee Biographical Questionnaire (EBQ) and Occupational Health Surveillance Questionnaire (OHRQ) by age groups as 22 - 31, 32 - 41, 42 - 51, 52 - 61 and 62+ and statistically analyzed. Length of working (LOW) showed close association with the duration of physical exercise (DPE) at adjusted R^2^ = 0.295 and type of work (TOW) at adjusted R^2^ = 0.598; and Education in the 62+ (oldest) age group. However such relationship was not observed in the 52 - 61 years age group even when DPE and Education were not significantly different from the 62+ group. In the 42 - 51 age group, significant correlation of LOW with DPE and TOW was found. Duration of physical activity could be an important factor associated with the duration of work in the oldest group. Type of work could be significant modifier in determining the length of working in older age-groups. Predecessor elderly groups might need to incorporate some of the measures that were significant in the oldest group, to improve their expectations to work longer. Larger studies could identify and capture various other measures that could be important both for the regional and national US perspective.

## 1. Introduction

Over the past decade, the duel impact of growth of aging population and rising health care costs have been adversely influencing many economies worldwide [1]; and projections show a looming negative trend for both aging population and costlier healthcare costs [2]. This predicament gets gravitated by the need of broader and expensive healthcare services as a result of years of chronic lifestyle linked with health risk behaviors in older individuals [3]. Notably, the relative age of the working population in all the developed countries has increased and at the same time the dynamics of global competitiveness have created the expectation for the aging employees to perform both qualitatively and quantitatively [4].

United States is experiencing a major demographic shift with growth of older population [5]. Older Americans, the most rapidly growing age group might not be meeting the expectation of being physically active [6]. Therefore anticipating, monitoring, and responding to changes in the work-life sustainability of employees are not only very important but also have critical consequences on social programs such as Medicare and Social Security in US [7]. Increasing costs of health care services in US also pose the question on identifying new approaches in an effort to prolong the duration of work, and simultaneously securing the medical coverage for the older employees [8].

Physical exercise has shown benefits for keeping employees physically able enough thereby contributing to extend their workability in studies involving the European population [9]. Importance of exercise testing and self-reported measures for the assessment of employment status has been reported earlier as well [10] [11]. One such study explored the role of modifiers of health and lifestyle that could support continuity of employment [12]. Lower education has also been associated with lower workability [13]. Another study has shown that the shift of work could have clinically significant manifestations in older employees [14], as well as importance of working hours [15]. Older employees with physical requirements at work could be exposed to overload than their younger peers [16]. However, there remains a gap in identifying definitive measures in US context. It appears that balancing the individual fitness and readiness, and requirements for work could be essential for continuing with the employment though there are no proper investigations done to identify any such.

Thus, we aim to identify the measures of physical fitness, specific health status, and lifestyle markers associated with the duration of work in the older US employees. These measures could serve as markers to the employees to tailor their efforts towards maintaining optimal health for sustaining workability [17]. Introducing such concepts could also support in developing viable strategies for reducing cost of escalating medical care costs, evidence based health management for extending workability, and a more involved geriatric work force [18].

## 2. Methodology

This study was approved by the Institutional Review Board of American University DC under investigational regulatory act for the participation of human subjects. Participants were approached at the academic and employment settings, and public locations in the greater Washington DC area to participate in this study. 40 male and female participants included in this study were non-treatment seeking, non-diagnosed otherwise healthy working individuals in the 22 - 75 years age range, and residing within United States territory. Children were not included, primarily because we did not focus on the late teenage and younger age groups. This study was open to all racial and ethnic groups.

In this study, data were primarily collected on demographics, education, and work and lifestyle activities. Responses were collected on a paper based questionnaire system using Customized-Employee Biographical Questionnaire (GRC453-EBQ [Version: Sep 2012]) and Occupational Health Surveillance Questionnaire (CG5187-OHRQ [Version: June 04]). These two validated questionnaires are well established and used in research for the past several years for evaluating socio-economic and health based parameters [19] [20]. Questionnaire sections with personal identification and medical information were omitted to avoid registering personal information. In the CG-OHSQ, following information were not collected: home address, “Potential Hazards”; and “Protective Equipment Worn”. Also part II of page 2, and Page 3 were not included in the data collection. However, “hobbies and active sports”, total time of exercise and type of physical activity, and in the “Work related experience” weight gain while working were asked. In EBQ, data collection did not include asking questions about the details on uniformed services details and personal data.

Responses were electronically coded for statistical analysis. For statistical analysis, “Length of Working” (LOW) measure (in yrs.) was evaluated as the primary dependent variable in this investigation. Data was collected for measures “Type of Work” (TOW, mental = 1, physical = 2, and both = 3), “Level of Training” (LOT; no exercise as 0, light as 1, moderate as 2, heavy as 3), “Duration of Physical Exercise” (DPE in hours)"Work Shifts” (WS; morning as 0, evening as 1 and night as 2)"Weight Increased” (WI, yes as 1 or no as 0). Above mentioned variables and age were evaluated for their inclusion as factors in this analysis.

Study sub-groups were distributed as 22 - 31, 32 - 41, 42 - 51, 52 - 61 and 62+ age ranges. Univariate ANOVA (UANOVA), multivariate ANOVA (MANOVA), and linear regression statistical models were used in statistical analyses. Between the groups analysis was performed using age-groups as factors. A power analysis was set at 0.25 with Cohen’s *d* of 84, which required a minimum of six participants in each group. Each of our study groups had eight participants in this study, thus sample sizes were sufficient in this pilot study. SPSS IBM version 22.0 (IBM, Chicago IL) and MS Office 2013 Excel application (MS Corp, Redmond WA) were used for data processing and analysis. Statistical analysis for this study was conducted at p ≤ 0.05.

This study was open to all US residents however, it was conducted in the greater Washington DC area and therefore region specific differences were not evaluated and participants were primarily local residents. This study was conducted as a pilot to identify the markers of work longevity, thus the sample size was small, which is a limitation in the study. Males and females in each age group were not in sufficient numbers to assess sex-based differences. Similarly, identifying any significant effect of race/ethnicity was not part of the study objectives.

## 3. Results

### 3.1. Demographics

The sample population was equally distributed in each group (Table 1). BMI was in overweight category in youngest (22 - 31 yr.) and two oldest groups (52 - 61 and 62+ yr.), however it was obese in the 42 - 51 yr. age group. Range of number of years of education was 6 to 24 in this study. Weight gain was observed in each group however largest was reported in the 42 - 51 yr. age group accompanying with lowest level of “Education”. Higher proportion of younger working individuals were involved in the evening or night shifts than their older cohorts.

### 3.2. Association of Length of Working with Health and Lifestyle Measures across the Age-Groups

**Figure 1:**
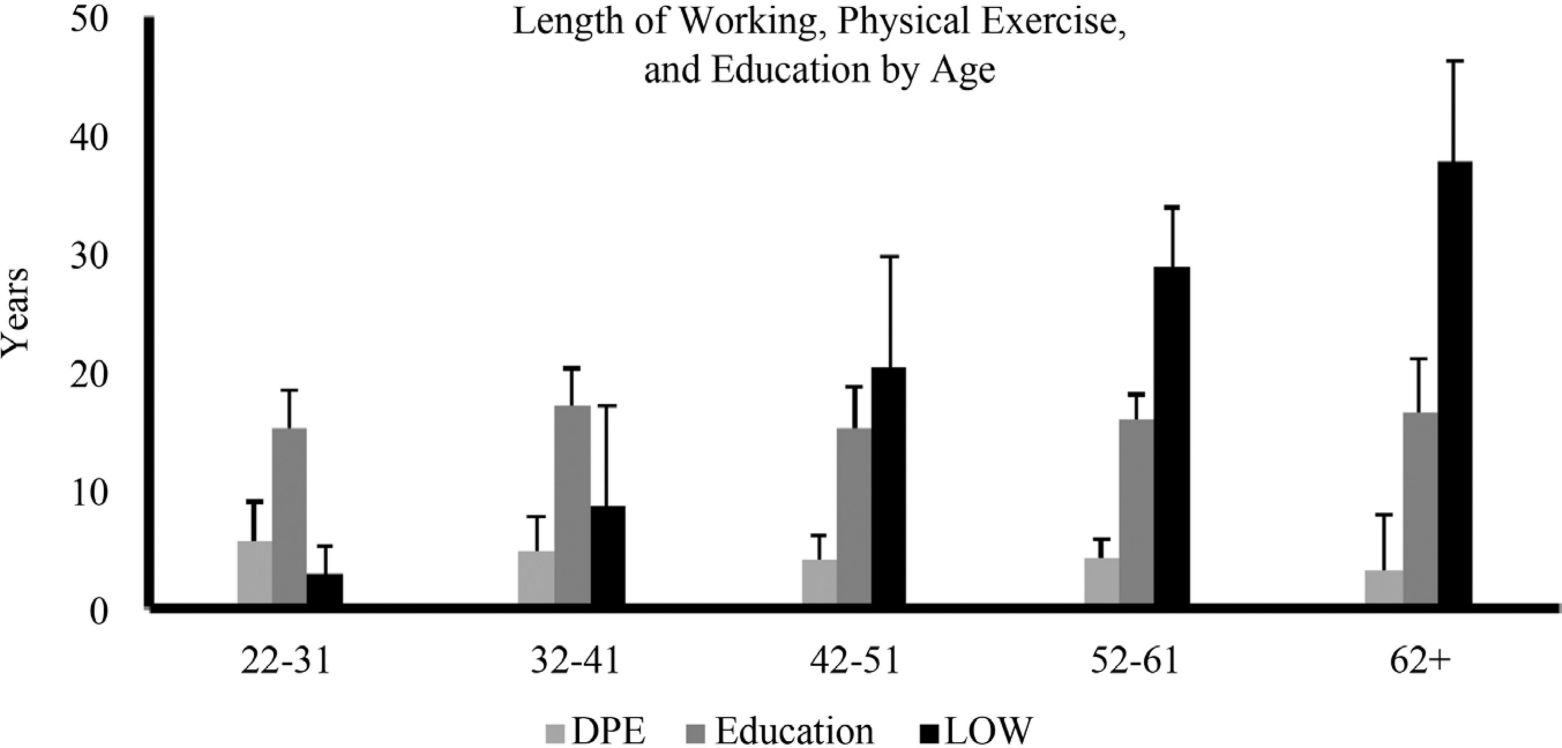
Health associated measures and length of working. Data for each measure presented for Duration of Physical Exercise as hours per week; Length of Working as years; and Education by age-groups as years. Data presented as Means ± Standard Deviation.

As anticipated the Length of working (LOW) measure showed progressive increase with age, with statistically significant difference across the groups, p ≤ 0.01. There was gradual lowering of duration of exercise (DPE) by the increasing order of the age group, with more number of hours of training performed by the youngest (5.8 ± 3.4) and least by the eldest groups (3.3 ± 2.6) (Figure 1).

We used MANOVA for evaluating LOW, DPE and Education across all the age-groups and TOW as factor. This analysis showed significant between-group TOW effects on LOW (p = 0.013, partial η^2^ = 0.283); and with Education (p ≤ 0.001, partial η^2^ = 0.447). Adding BMI or other factors did not change the analysis outcome. Such complex involvement of measures with between group analyses led to compare adjacent age groups to identify unique changes.

### 3.3. Comparison of Health and Lifestyle Measures between the Older Groups

We compared the difference in measures between the 62+ age group with the predecessor older groups individually. Analyzing the length of working in the 62+ age group with that of 52 - 61 age group, we found that there was a significant difference between the two groups (37.9 ± 8.5 vs. 28.88 ± 5.1 that was more than 10 years) partial η^2^ = 0.319, p = 0.023; and relatively more than any other consecutive group comparison. However there was no significant difference identified in the DPE or Education Measures between these two oldest groups. When we reviewed the LOW between 62+ and 42 - 51 age groups, we found anticipated significant difference, p = 0.002. However, there was no significant difference in the DPE or Education between the 62+ and 42 - 51 age groups. Inclusion of factors did not augment any of the between group analysis.

We did not report group difference analysis between 62+ and any other younger groups since large differences are anticipated due to age and experience gap.

### 3.4. Association of Length of Working in Older Groups

**Figure 2:**
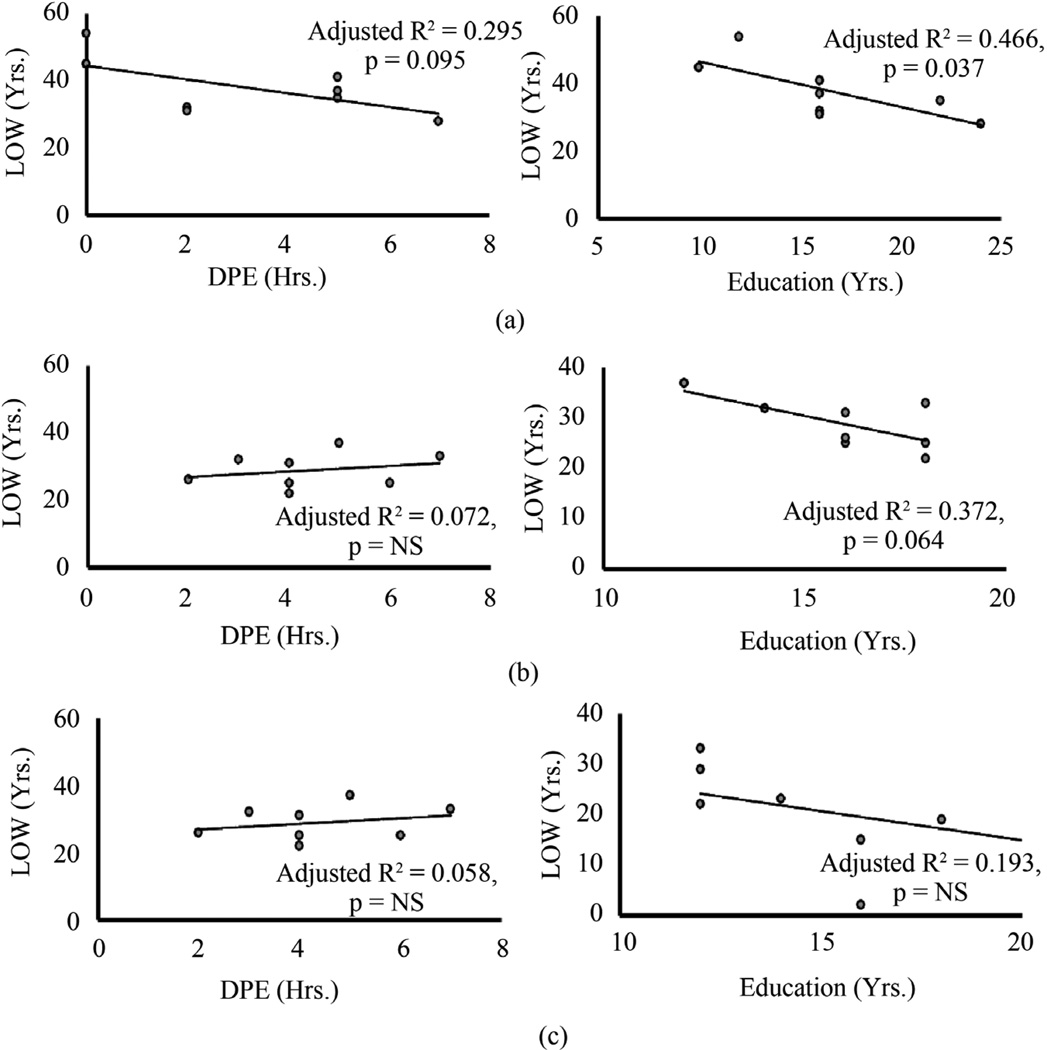
Relationship of length of working with duration of exercise and education in older age cohorts. (a) Length of Working with Physical Activity and Education in Age 62+ yrs. In the age cohort 62+, mild-to-moderate association observed between Length of working and Duration of Physical Exercise (This association augmented significantly with Type of Work as a factor). Significant moderate association was identified between LOW and Education. (b) Length of Working with Physical Activity and Education in Age 52 - 61 yrs. No significant association was found between Length of Working and Duration of Physical Exercise. Length of Working and Education showed mild association at borderline significance. (c) Length of Working with Physical Activity and Education in Age 42 - 51 yrs. There was no significant association determined between Length of Working and Duration of Physical Exercise; and Length of Working and Education. Statistical significance was set at p ≤ 0.05.

We evaluated the measures identified in the across the group analysis and between the older cohort analysis for the within 62+ group analysis. Physical exercise, DPE showed mild between-subjects effects with LOW, adjusted R^2^ = 0.295 at remote significance p = 0.095 (Figure 2(a)). However with TOW as a factor, this effect substantially augmented to adjusted R^2^ = 0.598 at significance, p = 0.060. Inclusion of other factor/s did not augment the significance any further. Such robust association albeit with lower significance could be attributed to the variability in the LOW data as evident by the standard deviation, none-the-less suggesting important role of DPE as a predictor, and TOW as a factor. “Education” measure showed moderate between-subjects effects on LOW, adjusted R^2^ = 0.466 at significance p = 0.037.

Identifying such characteristic changes in the oldest group, led us to look for the same changes in the predecessor groups. Hence, we evaluated two adjacent older groups (42 - 51 and 52 - 61) for within group analysis. In the 42 - 51 yr. age group, DPE did not show any significant effect on LOW (p = 0.565, partial R^2^ = 0.058), however with TOW as a factor, higher effect (partial R^2^ = 0.756) was observed at augmented significance p = 0.060 (Figure 2(c)). *Post-hoc* test showed underlying significance was attributing to the TOW variable “Physical” (sub-factor 2, p = 0.031). Education alone or with other factors did not show any significant effect on LOW. When we looked at the 52 - 61 yr. age group, there was no similar or statistically significant characterization as was found in the 62+ yr. age group (Figure 2(b)).

## 4. Discussion

Results of this study supported our primary aim that measures of physical fitness might play a significant role the longevity of work. Two of the measures of physical fitness; duration of physical exercise, and level of training were investigated; and duration of physical exercise demonstrated close relationship with workability. Evaluating the information on the level of training, it seems that the level of training tapered down when individuals have worked for a long time, as has been reported previously by others [21]. However, role of level of training could not be established to show any significant association. This could be attributed to the fact that working individuals may need to do physical exercise continuously; however, it might not be necessary to continue with the same intensity of exercise with the growing age. We did not find any other factors playing role especially BMI, which suggests that continuation of physical exercise might be more beneficial that the role of BMI. This is evident in US population since more than 65.7% of US population is overweight in 2001–2002 census [22]; however majority of US population is in workforce.

Our findings supported that the length of working is characterized by the modifiers that are specific health and lifestyle measures, type of work and education respectively. In fact, association of length of working and duration of physical exercise could only be characterized significantly with the type of work as a factor. Several association based studies have broadly demonstrated a greater than expected interrelationship between work and chronic conditions using health measures as modifiers [17]. This supports the fact that work-longevity might get significantly influenced by several aspects of lifestyle as modifiers. Some of the important measures identified in the oldest group did not show similar significant association within the predecessor older age groups. This disagreement could be important to understand that the developments in these two groups did not correspond with the oldest group or with the length of working within their own cohorts (Figure 2(b) and Figure 2(c)).

This is the first study of US workforce to evaluate factors that associate with the workability and longevity. Several such studies are being carried out in European and Asian regions to understand workability and aging [23]. However lack of significantly predictable measures could be a limitation in US for starting such investigations. Working individuals in younger and middle age-groups could get information for developing health planning to prolong working duration [24] from the association of measures that have shown significant association with longer work duration in some of the oldest working group. Physical exercise even of lighter form might be useful in maintaining a healthy lifestyle needed at work. Level of education and type of work could be additional contributing factors, which could support older age employees to continue with their employment. Sample of this pilot study is small, which is a limitation. Further regional effects could also be factor in this study; however, results from this study provide information for expanding such investigations towards larger population and adding more measures.

Even with the availability of healthcare and opportunities for education, US workforce is still facing chronic conditions as chief interference in their workability [25] [26]. Interestingly, older age at retirement is associated with lower risk of mental disorders namely, decreased risk of dementia and Alzheimer Disease [27] [28]. Loss of work ability and work life years could be much higher than anticipated in industrialized regions, where proportionally more physical labor is expected in the work force [29]. The lifestyle and physical exercise measures might not just be contributing to duration of work directly but also by maintaining optimal health [30]. Larger studies are needed to address this issue and identify further details that could be advantageous for determining the association with longevity of work-life in the older working population of United States.

## Figures and Tables

**Table 1:** Distribution of demographic measures by age groups. 42 – 51 years group showed highest increases in weight along with highest BMIs. Data presented as Mean with standard deviations (Mean ± Std).

Age groups (years) by participants	22 – 31 (8)	32 – 41 (8)	42 – 51 (8)	52 – 61 (8)	62 + (8)
Mean age (yrs.)	26.25 ± 2.4	35.6 ± 2.7	46.1 ± 2.3	54.75 ± 2.6	66.25 ± 3.0
Sex distribution (M = male, F = female)	4 M/4 F	5 M/3 F	3 M/5 F	5 M/3 F	3 M/5 F
Education (yrs.)	15.3 ± 3.2	17.1 ± 3.1	15.3 ± 3.5	16.0 ± 2.1	16.5 ± 4.6
Mean BMI category	28.2 ± 2.5	27.0 ± 2.6	31.3 ± 3.0	28.4 ± 3.3	28.7 ± 3.6
Weight increased (actual/total)	3/8	3/8	6/8	4/8	5/8
